# Practical Strategies Using Medical Cannabis to Reduce Harms Associated With Long Term Opioid Use in Chronic Pain

**DOI:** 10.3389/fphar.2021.633168

**Published:** 2021-04-30

**Authors:** Caroline A. MacCallum, Lauren Eadie, Alasdair M. Barr, Michael Boivin, Shaohua Lu

**Affiliations:** ^1^ Department of Medicine, Faculty of Medicine, Division of Community Internal Medicine, University of British Columbia, Vancouver, BC, Canada; ^2^ Department of Medicine, Faculty of Medicine, Division of Palliative Care, UBC, Vancouver, BC, Canada; ^3^ Faculty of Pharmaceutical Sciences, UBC, Vancouver, BC, Canada; ^4^ Department of Anesthesiology, Pharmacology and Therapeutics, Faculty of Medicine, UBC, Vancouver, BC, Canada; ^5^ CommPharm Consulting Inc., Barrie, ON, Canada; ^6^ Department of Psychiatry, Faculty of Medicine, UBC, Vancouver, BC, Canada

**Keywords:** harm reduction, opioid substitution, chronic pain, clinical tool, medical cannabis, morphine equivalent dose (MED), safety, cannabinoid based medicine (CBM)

## Abstract

**Background:** Chronic non-cancer pain (CNCP) is estimated to affect 20% of the adult population. Current United States and Canadian Chronic non-cancer pain guidelines recommend careful reassessment of the risk-benefit ratio for doses greater than 90 mg morphine equivalent dose (MED), due to low evidence for improved pain efficacy at higher morphine equivalent dose and a significant increase in morbidity and mortality. There are a number of human studies demonstrating cannabis opioid synergy. This preliminary evidence suggests a potential role of cannabis as an adjunctive therapy with or without opioids to optimize pain control.

**Methods:** In 2017, the Canadian Opioid Guidelines Clinical Tool was created to encourage judicious opioid prescribing for CNCP patients and to reevaluate those who have been chronically using high MED. Mirroring this approach, we draw on our clinical experiences and available evidence to create a clinical tool to serve as a foundational clinical guideline for the initiation of medical cannabis in the management of CNCP patients using chronic opioid therapy.

**Findings:** Following principles of harm reduction and risk minimization, we suggest cannabis be introduced in appropriately selected CNCP patients, using a stepwise approach, with the intent of pain management optimization. We use a structured approach to focus on low dose cannabis (namely, THC) initiation, slow titration, dose optimization and frequent monitoring.

**Conclusion:** When low dose THC is introduced as an adjunctive therapy, we observe better pain control clinically with lower doses of opioids, improved pain related outcomes and reduced opioid related harm.

## Introduction

Chronic non-cancer pain (CNCP) affects approximately 20% of the adult population, with 7% having chronic neuropathic pain (Andrew et al., 2014). CNCP is associated with poorer work-related outcomes, as well as compromised activities of daily living, worsened mental health, and higher use of the healthcare system (Andrew et al., 2014). Neuropathic pain is complex, due to the heterogeneity of its etiologies, co-existing symptom clusters and underlying mechanisms, and therefore poses significant challenges in its treatment and management (Bannister et al., 2020). The 2014 Canadian Pain Society (CPS) Consensus Statement for Chronic Neuropathic Pain recommends gabapentinoids, tricyclic antidepressants (TCAs) and serotonin/norepinephrine reuptake inhibitors as first-line treatments (Moulin et al., 2014). Opioids and tramadol are considered second-line, and are frequently used when first-line agents have failed (Moulin et al., 2014). Most agents for the management of neuropathic pain are used off-label (Goodman and Brett, 2019). They may have modest benefits, and are associated with a poorly tolerated adverse effect profile, especially in the context of analgesic polypharmacy, frequent in this population.

Although there is evidence to support the use of opioids for cancer-related pain, the evidence is limited for CNCP (Manchikanti et al., 2011; Reis-Pina et al., 2015). Opioids typically offer short-term analgesia similar to NSAIDs, TCAs, or nabilone (Busse et al., 2017). Opioids have a higher risk of side-effects, such as constipation, nausea, falls, confusion, hypogonadism, opioid-induced hyperalgesia, respiratory depression, sedation and addiction (Okoli et al., 2010; Lee et al., 2011; Boehnke et al., 2016; Busse et al., 2017; Jungquist et al., 2017). Although opioids are often used for CNCP conditions such as chronic low back pain and osteoarthritis, a 12-months randomized controlled trial did not show superior analgesia with opioids compared to non-opioid treatments (Krebs et al., 2018).

Opioid-use disorder, overdose, diversion, and escalating doses with opioid tolerance are associated with significant morbidity and mortality (Jones et al., 2015; Busse et al., 2017; Jones et al., 2018; Zhou et al., 2020). There is a strong correlation between opioid dose and the risk of harm, especially with regards to fatal overdose. The CDC reports that a 50 mg morphine equivalent dose (MED) doubles the risk of fatal overdose compared to 20 mg MED, and when the MED is greater than 90 mg the risk increases 10-fold (Center For Disease Control, 2017). In 2016 in the United States, 1.5% of all deaths, and 20% of all deaths in adults aged 24–35 years, were opioid-related, including prescription and illicit sources (Gomes et al., 2018). A Canadian study reported 1 out of every 550 patients prescribed chronic opioid therapy for CNCP died due to opioid-related causes (Kaplovitch et al., 2015).

United States and Canadian guidelines advise clinicians to carefully reassess risk-benefit ratio when opioid doses increase more than 50 mg MED in people initiating opioid therapy, and to avoid doses over 90 mg MED, as there is weak evidence for improvements in pain, but a significant increase in the risk of harm (Dowell et al., 2016; Busse et al., 2017). Clinicians face an increasing number of CNCP patients not reaching pain-management goals, and a lack of options that safely address their pain. Data has emerged showing the potential benefits of cannabinoids in chronic pain management. Cannabis is one of the least physiologically toxic analgesics, with a very high therapeutic index (Fitzgerald et al., 2013). This is likely due to paucity of cannabinoid (CB) receptors in the brainstem, and therefore lack of respiratory depression. As a result, cannabis may provide a safer alternative for some living with CNCP.

### Cannabinoids and Pain Modulation

Cannabinoids have multimodal mechanisms of action producing analgesia including: modulation of neuronal nociceptive processing, inhibition of pro-inflammatory molecule release, inhibition of mast cell activation, and modulation of endogenous opioid receptors in primary afferent pathways (Vučković et al., 2018; Amin and Ali, 2019; McKenna and McDougall, 2020). Similarly, cannabis may also provide relief for symptom clusters which accompany CNCP, such as nausea, anxiety, insomnia and depression via its effects on the endocannabinoid system (Manzanares et al., 2006; Hill et al., 2007) and so may also reduce the psychological distress associated with chronic pain (Feingold et al., 2017). These symptom clusters can be challenging to overcome using traditional pharmaceutical agents, which usually focus on a single symptom.

In the 2014 CPS Consensus Statement for Chronic Neuropathic Pain, cannabinoids were considered a third-line treatment option (Moulin et al., 2014). In this consensus, cannabinoids had a number-needed-to-treat of 3.4, in comparison to tricyclic antidepressants (TCAs) at 2.1, pregabalin at 4.5 and gabapentin at 6.5. Since that time, a number of clinical trials, systematic reviews and meta-analyses have reported that medical cannabis has moderate-substantial quality evidence for chronic pain and neuropathic pain in adults (Andreae et al., 2015; Ware et al., 2015; Yanes et al., 2019; MacCallum et al., 2021).

### Cannabinoids With Opioids for CNCP

Cannabis and opioids have been reported to offer synergistic analgesic effects when used concomitantly (Cichewicz, 2004; Abrams et al., 2011; Cooper et al., 2018). Utilizing adjunctive agents for pain relief is a well-recognized treatment approach, similar to the concomitant use of TCAs and gabapentinoids. Pre-clinical studies demonstrate that CB_1_ receptor antagonists can potentially reverse the peripheral anti-nociception induced by morphine in inflammatory pain models, indicating that endocannabinoid activity may be important for morphine’s actions (da Fonseca Pacheco et al., 2008). In a systematic review by Nielsen et al. (Nielsen et al., 2017), they observed that the preclinical evidence for interactions between cannabinoids and opioids was strong, with 17 of 19 studies confirming that synergistic effects from opioid and cannabinoid co-administration. A meta-analysis of the preclinical data showed that the median effective dose ED50 for morphine, when combined with THC, is 3.6 times lower than morphine alone. However, the evidence from clinical studies was notably less robust, as most studies were hampered by small sample size, and the lack of reporting on changes in opioid doses. Nevertheless, the evidence from existing clinical studies provides important preliminary data to show that there may be synergistic effects between cannabinoids and opioids; for example, in a study of 21 individuals with chronic pain, the addition of vaporized cannabis (3.56% THC) three times daily, significantly reduced pain by 27% (Abrams et al., 2011). Furthermore, the augmentation of the analgesic effect with vaporized cannabis did not significantly alter the opioid pharmacokinetics or plasma opioid levels, suggesting the combination does not increase the risk of serious opioid-related side-effects (Abrams et al., 2011; Reiman et al., 2017). This phenomenon can be described as *Cannabis-Opioid Mutual Potentiation* as cannabinoids interact and sensitize opioid receptors in anti-nociceptive brain regions (Cichewicz, 2004; Nielsen et al., 2017). The potential for synergistic side-effects between cannabinoids and opioids will require further study, although there is currently little evidence for these sequelae. A recent naturalistic/observational study of medical cannabis users at a clinic, where six hundred opioid-using patients were monitored over six months by physicians, observed that by the end of the observational period, 26% of clients had stopped using opioids, while an additional 55% had reduced their average opioid dose by 30% (Rod, 2019). Clearly, these types of pilot studies provide promising evidence of cannabinoid/opioid synergies, but large scale, well controlled clinical trials will ultimately be needed to confirm these effects (Le Foll, 2021).

There is preliminary but promising data to support that medical cannabis initiation can help reduce the opioid dose required to produce pain relief, or substitute the use of opioids altogether, which could help reduce the risk of fatal opioid-related overdose (Lucas and Walsh, 2017; Reiman et al., 2017; Knerich et al., 2019), although this has not been reported in all studies (Merlin et al., 2019). A cross-sectional retrospective survey reported that medical cannabis use was associated with a 64% decrease in opioid use, improved quality of life, and fewer medication-related side-effects (Boehnke et al., 2016). Two recently published studies using Medicaid program data from 2011 to 2016 in the United States, support that states where cannabis was legalized had lower opioid prescribing (Bradford et al., 2018; Wen and Hockenberry, 2018). Specifically, there was a 5.88% lower rate of opioid prescribing in states with medical cannabis laws, and 6.38% in states with both “adult-use” and medical laws (Wen and Hockenberry, 2018). States with medical cannabis laws had a 24.8% lower mean annual opioid overdose mortality rate compared with states without medical cannabis laws (Bachhuber et al., 2014).

### Initiating a Medical Cannabis Adjunct Trial

Preliminary clinical data indicates a role for cannabis as an adjunct to opioids for the management of chronic pain. In Table 1 we propose a cannabis titration trial to model the currently recommended clinical approach to an opioid trial, suggested by Canadian opioid CNCP guidelines (Busse et al., 2017). This allows for cautious initiation of cannabis, with a focus on efficacy, safety, titration and monitoring.

**TABLE 1:** Proposed steps for cannabis adjunct initiation trial with opioid therapy.

Step 1—Assessment
Step 2—Starting cannabis at a low dose
Step 3—Slow titration until optimal dose
Step 4—Frequent monitoring
Step 5—Optimizing the titration
Step 6—Consider stopping the trial and discontinuing cannabis (if no response)

#### Step 1—Assessment

We recommend cannabis be considered for individuals who are not reaching pain management goals consuming ≥90 mg MED, with a weaker recommendation for 50–90 mg MED per day. This opioid dose is cited in guidelines where the risks of further dose increases exceed the benefits (Busse et al., 2017). Clinicians are encouraged to discuss the risks and benefits of cannabis, similarly to any new medication initiated with their patients. It would be advisable to assess and document the patient’s baseline pain control using a validated tool as a baseline (e.g., Brief Pain Inventory) and to screen for risk-factors for cannabis use disorder, prior to initiating cannabis therapy. In addition, each medical professional should discuss the potential of THC impairment, including the additive pharmacodynamic interactions with additional sedating/impairing substances medications such as alcohol, benzodiazepines, non-benzodiazepine Z-drugs, TCAs and anti-epileptics (Lucas et al., 2018).

It is important for clinicians to discuss the potential impact of cannabis on driving. General recommendations for driving after cannabis use state that the patient should wait at least 4 h after inhalation, 6 h after oral ingestion and 8 h if euphoria is experienced (College of Family Physicians of Canada, 2014). The daily low-dose THC utilized by medical cannabis patients promotes tolerance to the negative side-effects, as noted in a recreational cannabis study showing less neurocognitive and motor impairment with frequent (>4 times per week) cannabis users compared to infrequent users at the same dose of THC (Nurmikko et al., 2007; Ramaekers et al., 2009; Desrosiers et al., 2015). Therefore, impairment associated with medical cannabis use should be considered differently from recreational use (Eadie et al., 2021). It is recommended that physicians be familiar with impaired driving legislation from government as well as provincial/state bodies, social and occupational considerations (Heath Canada, 2016).

Assessment of personal and family history of mental health illness is important for clinicians to take into consideration when initiating and increasing the dose of THC. Clinicians can monitor for changes or worsening of psychological/cognitive functioning which are THC dose dependent (e.g., difficulties in concentration and memory (Kroon et al., 2020; McCartney et al., 2021), or psychotic symptoms) (Alexander et al., 2017; Gicas et al., 2020; Jones et al., 2020). Another important patient population to consider are youth. Younger age of first cannabis use is associated with earlier onset of schizophrenia and bipolar disorder in those who are predisposed (Murray et al., 2017). In youth under 18 years of age who use cannabis regularly, there may be increased risk of persistent cognitive effects, increased social dysfunction, anxiety and/or depression (Degenhardt et al., 2013; Gobbi et al., 2019; Tucker et al., 2019). With these considerations, only clinicians with expertize in youth and medical cannabis should be involved in these cases. This tool is not intended for youth; further research in this area is necessary.

Overall, we encourage clinicians to optimize biopsychosocial modalities including mindfulness, yoga, therapeutic movement, nutrition, cognitive behavioral therapy, physiotherapy or occupational therapy or other modalities which may help enhance neuroplasticity, promote patient participation, resilience and self-management for the best possible long term outcomes.

We also recognize that comprehensive assessments for addiction risk and psychological dependency are integral part of any long-term use of opioid or cannabinoid for the treatment of pain. The focus of this document is to mitigate the harms associated with long term opioid use. The strategies outlined should reduce the risk associated with chronic opioid pain management; the aim is to reduce overall opioid requirement, particularly those on high MED. We recognize the strategies outlined are one part of full spectrum care and are not meant to be a single substitute for all medications or opioids. We recognize the inherent addiction risk associated with cannabinoid use and those with other substance use disorder are at increased risk of cannabis use disorder (Liu et al., 2018). We recommend for all patients, including those without a history of substance use disorder, to have ongoing monitoring for addiction risk with instruments such as such as the Opioid Risk Tool (Webster and Webster, 2005) and Cannabis Use Disorder Identification Test (Adamson et al., 2010).

#### Step 2—Starting Cannabis at a Low Dose

For most accurate dosing, we propose the ingestion of cannabis, which allows more precise dosing and is associated with less potential respiratory harm when compared to inhaled cannabis (Fischer et al., 2017). Cannabis oils may provide up to 6–8 h of symptom relief as a result of the conversion of THC to 11-OH-THC by the liver (MacCallum and Russo, 2018). For inflammatory pain, such as autoimmune conditions or inflammatory arthritis, we advise starting with a chemovar (strain) containing predominantly CBD for daytime use. From clinical experience and anecdotal evidence, CBD is associated with a lower adverse effect profile (e.g., low risk of dysphoria or impairing effects – including both psychotomimetic and cognitive effects), and may be sufficient for inflammatory pain without the addition of THC. We recommend an initial starting dose of 5–10 mg of CBD, 1–2 times daily (MacCallum and Russo, 2018). For patients with CNCP who have difficulty sleeping, or pain at night, we suggest a starting dose of 2.5 mg of THC in the evening (MacCallum and Russo, 2018). An evening dose (vs. daytime) allows the patient to minimize potential side-effects. If the patient is elderly, has complex comorbidities, or extensive polypharmacy, clinicians may consider a lower starting dose of 1–1.25 mg of THC. Starting cannabis treatments at a low dose is consistent with the medical guidelines for treatment with Sativex® (nabiximols) (which is a natural cannabis extract of approximately equal concentrations of THC and CBD), in which treatment begins with the lowest full dose possible (Product Monograph: Sativex, 2012).

#### Step 3—Slow Titration Until Optimal Dosing

For individuals using a THC-based product (including a balanced 1:1 THC:CBD chemovar), we suggest a dosage increase between 1–2.5 mg of THC every 2–3 days, starting first with the evening dose (as above) (MacCallum and Russo, 2018). This is similar to the initiation of some other new medications (Flack et al., 2000; Cahn and Cefalu, 2016; Yin et al., 2017; Yuen et al., 2018), such as drugs for treating hypertension, hyperglycemia and psychosis, where the approach is to start with a lower dose, and monitor closely for side-effects so that the dose can be safely increased over time. We therefore suggest starting cannabis at a low dose and titrating slowly (Lucas et al., 2018; MacCallum and Russo, 2018). Using this protocol, THC should be titrated until the lowest effective dose is reached, minimizing side-effects and therefore providing the greatest therapeutic effect. This approach is also consistent with the official treatment guidelines for the use of Sativex® (Product Monograph: Sativex, 2012), where the dose increases gradually over time as side-effects can be assessed. It is important to remind patients that the dose is increased until the patient reaches their treatment goal. They should not continue to titrate a THC product until they feel euphoria. If the patient has residual daytime symptoms, clinicians may consider adding in a daytime dose of THC if already using CBD or 1:1 THC:CBD ratio product, and titrating in a similar fashion to the evening dosing. Some patients may require up to three doses of cannabis oil per day to control symptoms. We generally recommend a maximum daily dose of 30 mg of THC (MacCallum and Russo, 2018); however, if the patient is responding well up to that point, it may be worth continuing to increase until maximal symptom relief, as long as side effects are not outweighing the picture. If there is no response by 30 mg, we do not recommend going higher.

While high dose CBD with minimal THC content has seen a surge of recent use for medical conditions such as refractory epilepsy and psychosis (McGuire et al., 2018; Bitencourt et al., 2021), there is little-to-no evidence to suggest that CBD alone can provide the pain control necessary to allow for stabilization or reduction of opioid doses; studies on this topic have tended to be statistically underpowered or lacking appropriate placebo control groups (Sholler et al., 2020). Nevertheless, among individuals with an opioid dependence disorder, CBD may be beneficial as a treatment for harm reduction, independent of any effects on pain. While this issue is beyond the general scope of this dissertation, it is worth noting that a well-controlled study reported that 400 and 800 mg of CBD were effective in decreasing cue-induced drug craving and anxiety in abstinent heroin users (Hurd et al., 2019). Studies have confirmed that at doses below 1500 mg of CBD, the drug does not produce hedonic effects or cognitive impairment (Birnbaum, 2019). Nevertheless, these high CBD doses may be associated with a few side effects; including gastrointestinal, sedation and - most importantly - there is increasing evidence that sustained high doses of CBD may result in transaminase elevations which may represent drug-induced liver injury (Brown and Winterstein, 2019; Watkins et al., 2020).

The majority of cannabis-related side-effects are due to the THC content and are dose-dependent. Naïve cannabis users without tolerance to THC may be more prone to experience side-effects. The most common acute symptoms include dizziness, drowsiness, anxiety and euphoria. While preclinical studies have provided mixed findings on whether treatment with THC can result in weight gain in animals (Cluny et al., 2015; Assa-Glazer et al., 2020), the most comprehensive epidemiological study to date in humans indicated that cannabis users were less likely to gain weight than individuals who have never used cannabis (Alshaarawy and Anthony, 2019), and so this should not be considered a common side-effect of cannabis for most users, despite the common perception of cannabis use causing the “munchies” (Sansone and Sansone, 2014). These side-effects are mitigated when patients start with lower doses and wait an appropriate amount of time before increasing their dose (i.e., “start low and go slow”). Tolerance to side-effects normally develops a few days after each new dose increase. With each increase, the clinical response and potential side-effects should be reassessed. If adverse effects are experienced, the dose should be reduced to the previously tolerated dose, and a slower titration should be undertaken. From clinical experience, the most effective way to reduce side-events is to use CBD-predominant chemovars during daytime, oils (which have a longer duration of action compared to inhaled routes), and reserving THC-containing strains to evening dosing when possible. For CBD-predominant products, we suggest an increase of 5–10 mg every 1–2 days, titrating to a minimum target of 50 mg daily before suggesting patient is a non-responder. Clinicians may consider higher CBD doses if the patient is partially responding.

#### Step 4—Frequent Monitoring

During the initial titration phase, we encourage clinicians to reassess patients in 2–4 week intervals to monitor for side-effects, patient response, as well as patient education and support. An important component to assessing the risk vs benefit for a patient is to monitor their symptom response with cannabis use. A dosing log can be useful to track product details and to determine if symptoms are improving or worsening over time (MacCallum, 2021). Once patients have achieved a therapeutic effect, follow-up may be less frequent (every 3–6 months), similar to follow-up for other analgesic medications (Moulin et al., 2014). We strongly recommend the involvement of family members and other allied healthcare providers to monitor for efficacy and side-effects, and encourage biopsychosocial strategies.

Clinicians are encouraged to use a validated pain tool as recommended in Step 1 to assess and document treatment response. Additionally, we encourage use of questionnaires such as the Generalized Anxiety Disorder Assessment (GAD)-7 for anxiety (Spitzer et al., 2006) and the Patient Health Questionnaire (PHQ)-9 for depression (Kroenke et al., 2001), which frequently occur in this patient population (Yau et al., 2019). The current CNCP opioid treatment guideline includes the use of urine drug screens to monitor both compliance and risk of addiction. The judicious use of urine drug screens should be a part of the standard of long-term opioid and cannabis treatment monitoring. For clinicians who have a larger patient population, additional training and education on cannabis is strongly recommended, as this is an evolving field.

#### Step 5—Optimizing the Titration

For patients using CBD-dominant oil, who reach a daily dose of 50 mg and are not achieving pain management outcomes, we advise clinicians to consider the addition of 2.5 mg of THC at bedtime, and during the day, if appropriate. They should maintain on the same dose of CBD, while slowly titrating THC as recommended in step 3. Although side-effects generally dissipate within 24–48 h, if symptoms persist or increase in intensity, we recommend decreasing THC and/or increasing CBD (MacCallum and Russo, 2018).

Evidence suggests THC is helpful for neuropathic pain control (Abrams et al., 2011; Geoffrion et al., 2020; Rouhollahi et al., 2020). For treatment-resistant neuropathic pain, we advise considering 1:1 balanced THC:CBD cannabis oil, as the literature suggests the addition of CBD may mitigate THC-related side-effects (Lucas and Walsh, 2017). These findings hold true in our clinical practice. “Breakthrough” pain may be best addressed using inhaled, vaporized THC, which has onset of action within 5–10 min and duration of action of approximately 2–4 h. This is due to rapid absorption of THC from the lungs into the bloodstream, without conversion to 11-OH-THC by the liver (Huestis, 2007) (unlike THC oil). Bearing in mind, vaporized or inhaled concentrates of THC may have more intense psychological or cognitive side-effects. Additionally, non-legal sources of nicotine or cannabis “vape pens” are not regulated by health authorities; consequently, some supplies contain solvents, diluents, thickeners and fillers in their formulations which have been associated with lung disease (Layden et al., 2020).

Topical cannabis preparations are another option, which are ideal for localized symptoms (Alward, 1998; Maida and Corban, 2017; Highet et al., 2020; Xu et al., 2020). Onset is usually delayed relative to smoking or vaping (Huestis, 2007), with variable duration of action; however, evidence is limited compared to ingested or inhaled cannabis (Grotenhermen, 2003). Topicals may be helpful for those who do not want to take THC oil during the daytime to minimize potential impairment during work, or when driving (Fischer et al., 2017). Comparable to current opioid initiation guidelines for CNCP, a successful trial and optimal dose is achieved when a patient reports ≥30% decrease in pain intensity using a validated tool and/or an improvement in overall function.

#### Step 6—Consider Stopping the Trial and Discontinuing Cannabis if No Response

If the patient reaches a total daily dose of ≥30 mg of THC without an adequate response, we advise clinicians to consider the patient as a “non-responder”. Prior to reaching 30 mg of THC monotherapy, we would expect a trial of daytime CBD. Based on our extensive clinical experiences, despite the lack of reported evidence in the literature, we encourage clinicians to taper cannabis by decreasing the dose of THC by 2.5–5 mg every 2–3 days until discontinuation. Slower titration can be considered if the patient experiences significant cannabis withdrawal effects (e.g., insomnia, anxiety, increased pain); however, this is not typical if using the proposed taper and dosing regimen outlined above. Withdrawal is usually seen with more regular THC dosing (Struble et al., 2019; Bahji et al., 2020), and more commonly in recreational populations.

## Conclusion

Preliminary evidence suggests a potential role for cannabis as an adjunct to opioids for the management of CNCP. Opioid-related harm is dose-dependent. Cannabis adjunct therapy may allow clinicians to use lower opioid doses and reach better pain management outcomes, without an exposure to the equivalent level of risk and harm. We propose a cannabis titration trial which mimics the steps for an opioid trial as per the 2017 Canadian Guideline for Opioids for CNCP (Busse et al., 2017). Based on current research evidence and clinical experience with this group, the outlined approach will allow clinicians to manage cannabis with an emphasis on efficacy and safety.

There are several limitations to the proposed model. Firstly, it is based in part on the present summary of the extant literature – which focuses on key studies while integrating clinical experience, rather than via systematic review, as we have conducted previously to determine the effects of drugs (Tse et al., 2012; Tse et al., 2014; Whitney et al., 2015). Additionally, it is not possible to determine which sub-group of opioid-dependent patients will benefit from the addition of cannabis. Pharmacokinetic studies indicate that people administered cannabis can show an extremely large range individually in how the different cannabinoids may be absorbed and metabolized in different parts of the body (Huestis, 2007; Foster et al., 2019), rendering predictions of individual clinical response challenging. Future developments in areas such as pharmacogenomics, which show increasing success in predicting both therapeutic and side-effects of drugs (Hryhorowicz et al., 2018; Bousman et al., 2021), represent areas for study to help identify which opioid users would most benefit from cannabis therapy.

There is also no clear consensus on the optimal doses of cannabis, namely CBD and THC, required to achieve optimal pain control in patients using opioids. The clinical landscape of cannabinoid-based therapies is complex, as it includes a diverse range of cannabis-related products, such as different cannabis chemovars, pharmaceutical products (e.g., Sativex® and nabilone) and CBD from both cannabis and hemp origins. All of these may potentially interact with other medications as well (Gottschling et al., 2020). Treatment algorithms must therefore encompass all of these individual medications and a manageable way, requiring some degree of flexibility. Finally, the proposed model is largely based on clinical experience and is not fully supported by randomized control trials. However, the underlying strategies which create the foundation for this model are based on extant cannabis research, including cannabis-opioid synergy and cannabis efficacy in neuropathic pain. This clinical tool is based on a model of continuing evaluation with major focus on education and risk assessment. This tool represents an opioid-dose de-escalation strategy in CNCP patients who are taking >50 mg MED higher, and so an important future goal will be to develop an effective opioid-tapering strategy once optimal cannabis dose (and symptoms) has been achieved.

## Data Availability

The original contributions presented in the study are included in the article/Supplementary Material, further inquiries can be directed to the corresponding author.
